# Performance of 3 Sets of Criteria for Potentially Inappropriate Prescribing in Older People to Identify Inadequate Drug Treatment

**DOI:** 10.1001/jamanetworkopen.2022.36757

**Published:** 2022-10-20

**Authors:** Susanna M. Wallerstedt, Staffan A. Svensson, Johan Lönnbro, Fredrik Hieronymus, Johan Fastbom, Mikael Hoffmann, Naldy Parodi López

**Affiliations:** 1 Department of Pharmacology, Sahlgrenska Academy, University of Gothenburg, Gothenburg, Sweden; 2 HTA-Centrum, Sahlgrenska University Hospital, Gothenburg, Sweden; 3 Närhälsan Hjällbo Health Center, Gothenburg, Sweden; 4 Department of Internal Medicine, Sahlgrenska University Hospital, Gothenburg, Sweden; 5 Aging Research Center, Department of Neurobiology, Care Sciences and Society, Karolinska Institutet and Stockholm University, Stockholm, Sweden; 6 NEPI Foundation–Swedish Network for Pharmacoepidemiology, Linköping University, Linköping, Sweden; 7 Närhälsan Kungshöjd Health Center, Gothenburg, Sweden

## Abstract

**Importance:**

Potentially inappropriate medications (PIMs) and potential prescribing omissions (PPOs) are used in research to reflect the quality of drug treatment in older people and have been suggested for inclusion in core outcome sets for evaluation of interventions for improved prescribing. Their validation so far, however, is primarily restricted to expert opinion–based processes.

**Objective:**

To evaluate the performance of 3 explicit PIM/PPO criteria sets as diagnostic tools to identify inadequate drug treatment in older patients.

**Design, Setting, and Participants:**

This diagnostic study analyzed patients aged 65 years or older consecutively included from 2 primary health care centers from October to November 2017. Data were analyzed from February to August 2022.

**Exposures:**

The PIMs/PPOs were concordantly identified by 2 specialist physicians (2018-2019) retrospectively after a planned physician visit, using 3 European PIM/PPO criteria sets and without knowledge of this diagnostic study.

**Main Outcomes and Measures:**

Area under the receiver operating characteristic (ROC) curve, reflecting the ability of PIM/PPO criteria sets to identify the reference standard of inadequate drug treatment, determined by 2 specialist physicians in consensus. Inadequate drug treatment implied that additional action related to the medication could be medically justified before the next regular visit.

**Results:**

A total of 302 patients were analyzed (median age, 74 [IQR, 69-81] years; 178 women [59%]; median number of drugs in the medication list, 6 [IQR, 3-9]); 98 patients (32%) had inadequate drug treatment. A total of 0 to 8 PIMs/PPOs per patient were identified using the Screening Tool of Older Persons’ Prescriptions (STOPP)/Screening Tool to Alert to Right Treatment (START) criteria, 0 to 6 with the European EU(7)-PIM list, and 0 to 12 with the Swedish set of indicators of prescribing quality. The areas under the ROC curve for the 3 sets to identify the reference standard for inadequate drug treatment were 0.60 (95% CI, 0.53-0.66) for the STOPP/START criteria, 0.69 (95% CI, 0.63-0.75) for the EU(7)-PIM list, and 0.73 (95% CI, 0.67-0.80) for the Swedish set. For comparison, the area under the ROC curve was 0.71 (95% CI, 0.65-0.78) using the number of drugs in the medication list.

**Conclusions and Relevance:**

In this diagnostic study, the evaluated PIM/PPO sets had poor to fair performance as diagnostic tools to identify inadequate drug treatment, comparable with a simple count of the number of drugs in the medication list. These findings suggest that use of PIMs/PPOs as indicators of drug treatment quality in core outcome sets for the evaluation of interventions for improved prescribing may need reconsideration.

## Key Points

**Question:**

Can criteria for potentially inappropriate medications (PIMs) and potential prescribing omissions (PPOs) be used to identify inadequate drug treatment in older people?

**Findings:**

In this diagnostic study, receiver operating characteristic (ROC) curves for 3 PIM/PPO criteria sets showed poor to fair performance comparable with merely counting the number of drugs.

**Meaning:**

The findings of this study suggest that using these PIM/PPO criteria sets as indicators of drug treatment quality, and within core outcome sets for the evaluation of interventions aimed at improved prescribing, may not provide valid results; the tools misclassify a substantial proportion of patients, both those with inadequate and those with adequate drug treatment.

## Teaser

This diagnostic study examined the use of 3 tools to identify inadequate drug treatment in older adults.

## Introduction

The scientific field of measuring prescribing quality expanded in the late 20th century, as exemplified by the introduction of the Beers criteria.^1,2^ A pivotal meeting took place in 2004, attended by 40 experts from 19 European countries, the US, Canada, and Australia.^3^ This initiative led to the recommendation that indicators of prescribing quality should be developed, ie, measurable elements of prescribing performance for which there is evidence or consensus that they reflect the quality and, hence, change in quality, of provided health care.^3^ Since then, potentially inappropriate medications (PIMs) and potential prescribing omissions (PPOs) for older people, according to specified sets of criteria (ie, tools), have been increasingly used as a means to describe the quality of drug treatment in this age group.^4,5,6^ It has been suggested that PIMs/PPOs should be included in core outcome sets for the evaluation of interventions aimed at improved prescribing.^7,8,9^ Examples of tools to identify PIMs/PPOs are the Screening Tool of Older Persons’ Prescriptions (STOPP)/Screening Tool to Alert to Right Treatment (START),^10^ the European (EU)(7)-PIM list,^11^ and a Swedish set of criteria for older patients developed by Sweden’s National Board of Health and Welfare.^12^

Validity evidence in support of PIM/PPO criteria to reflect quality of drug treatment in older people has, however, so far primarily been limited to content validity, showing the extent to which participants in expert opinion–based processes, typically using Delphi methods to concur in definitions of PIM/PPO criteria.^10,11,12,13,14,15^ However, evidence of concurrent validity showing to what extent the indicators actually measure what they claim to measure is comparatively sparse. To our knowledge, there are few studies addressing the clinical relevance, and a substantial proportion of the PIMs/PPOs has been reported not to be relevant for the individual patient.^16,17^ Furthermore, a systematic review concluded that there is an abundance of drug-oriented listing approaches, but that much is still lacking regarding clinical validation.^18^ Because there is no universally accepted standard to determine the quality of drug treatment, however, such validation may be a challenge. To bypass the fact that evidence-based guidelines tend to map poorly to complex multimorbidity,^19^ such a standard would preferably apply an overall medical perspective taking into account the need for priorities in health care.

Receiver operating characteristic (ROC) curves are the usual way of evaluating diagnostic tests against a standard^20^ but, to our knowledge, ROC curves have not previously been used to assess the ability of PIM/PPO criteria to discriminate patients with inadequate drug treatment from those with adequate drug treatment. Such an approach could add valuable insights regarding the performance of the criteria sets to identify inadequate drug treatment over a range of possible cutoff points.^21^ Often several PIMs/PPOs are identified in a single patient^22^ and ROC curves are widely used to determine appropriate cutoff points.^23^ In this study, we aimed to evaluate the performance of 3 sets of PIM/PPO criteria as diagnostic tools to identify inadequate drug treatment in older patients.

## Methods

In this diagnostic study, we used data from 2 previous studies investigating the association between recorded medication reviews and adequacy of drug treatment, as well as the clinical relevance of PIMs/PPOs, in 302 consecutive patients aged 65 years or older attending a planned physician consultation in 1 of 2 primary care centers in 2017.^16,24^ Data were obtained in October to November 2017. Data analysis was conducted from February to August 2022. The study was approved by the regional ethical review board in Gothenburg, Sweden. Informed consent was waived by the board because the benefit-risk balance was assessed as positive, given the scientific value of the study and the integrity risk. This study followed the Standards for Reporting of Diagnostic Accuracy (STARD) reporting guideline.

A physician specialist in clinical pharmacology and family medicine (S.A.S.) and a physician specialist in family medicine (N.P.L.) independently identified PIMs/PPOs according to 3 comprehensive sets of explicit PIM/PPO criteria: the STOPP/START criteria, version 2, including 80 PIMs and 34 PPOs^10^; the EU(7)-PIM list including 282 PIMs^11^; and a Swedish set of indicators of prescribing quality including 87 PIMs and 22 PPOs.^12,25^ The assessors’ identification of PIMs/PPOs was performed to systematically ascertain that important aspects of potentially inappropriate prescribing were not overlooked before the overall assessment of adequacy of drug treatment. All PIMs/PPOs concordantly identified by both assessors were included in the main analyses of the present study.

As reference standard, the same 2 specialist physicians determined in consensus, after independent assessments, the adequacy of drug treatment for each patient. The drug treatment was considered inadequate if action related to the medication would have been medically justified before the next regular consultation.^24^ If not, the drug treatment was considered adequate. In the assessments, an overall medical perspective was applied, taking the individual patient’s state of health into account.

All assessments were performed retrospectively (2018-2019). The assessors were blinded to the issue of interest in the present analysis; they were not aware that the performance of PIM/PPO tools as diagnostic tools was to be evaluated. They used printouts of electronic medical records over the 2½ years preceding this visit, including laboratory test results, hospital discharge records, vaccinations, prescriptions, and interaction alerts integrated into the medical record system.

### Statistical Analysis

Statistical analyses were conducted using SPSS Statistics for Windows, version 27.0 (IBM Corp). Area under the ROC curve was calculated, including the 95% CI, for PIMs/PPOs according to the 3 criteria sets to identify the reference standard for inadequate drug treatment. We analyzed the full criteria sets, as well as the PIM and PPO criteria sets separately, because both alternatives are used in the scientific literature.^4,5,6^ We interpreted the accuracy of the diagnostic tests based on the ROC curve; 0.60 was considered poor, 0.70 fair, 0.80 moderate, 0.90 high, and 0.95 near perfect.^20^ Because a previous study^26^ had omitted 2 STOPP criteria and 2 START criteria concerning implicit rather than explicit PIMs (ie, any drug prescribed without an evidence-based clinical indication or beyond the recommended duration, where treatment duration is well defined), as well as PPOs regarding influenza and pneumococcal vaccines, we likewise performed our analyses without these criteria. In addition, we estimated the optimal cutoff point (number of PIMs/PPOs identified) for each criteria set to discriminate between adequate and inadequate drug treatment, by identifying the highest sum of the sensitivity and specificity.^27^ At these exploratory optimal cutoff points, we also calculated the positive predictive value and negative predictive value as well as the positive and negative likelihood ratios. For comparisons, we used the number of drugs in the medication list, including regular drugs and drugs prescribed as needed, as a diagnostic tool. This comparator was chosen because the number of drugs is readily calculated and because polypharmacy has been used as an outcome measure in interventional studies for improved prescribing.^28^

Interrater agreement regarding the independent assessments of adequacy of drug treatment preceding the consensus decision was evaluated with κ statistics. Because binary outcomes may mask uncertainties in the reference standard,^29^ 2 sensitivity analyses were performed. First, we considered the drug treatment inadequate if both specialist physicians independently had made this decision. Second, we only required 1 assessor to have determined the drug treatment as inadequate.

To take into account the issue of discordant identification of PIMs/PPOs,^16^ we performed additional sensitivity analyses, including all PIMs/PPOs identified by either of the assessors. For the latter, as well as for concordantly identified PIMs/PPOs, we also calculated the sensitivity, specificity, and positive and negative predictive values, with cutoff points predetermined as greater than or equal to 1 PIM/PPO and greater than or equal to 5 drugs in the medication list, the latter being the most common cutoff point for polypharmacy.^30^

## Results

The patients were aged 65 to 99 years (median age, 74 [IQR, 69-81] years); 178 were women (59%), 124 were men (41%); and they had 0 to 20 drugs in the medication list (median, 6 [IQR, 3-9] drugs) (Table 1). The assessors concordantly identified 1010 PIMs/PPOs in 259 patients (86%) and, according to our reference standard, the drug treatment was categorized as inadequate in 98 patients (32%). A total of 0 to 8 PIMs/PPOs per patient were identified using the STOPP/START criteria, 0 to 6 with the EU(7)-PIM list, and 0 to 12 with the Swedish set. Common PIMs/PPOs, and aspects related to inadequate drug treatment, are described in eTable 1 in the Supplement. The areas under the ROC curve for the complete sets to identify the reference standard for inadequate drug treatment were 0.60 (95% CI, 0.53-0.66) for the STOPP/START criteria, 0.69 (95% CI, 0.63-0.75) for the EU(7)-PIM list, and 0.73 (95% CI, 0.67-0.80) for the Swedish set (Table 2, Figure). Regarding the independent assessments of drug treatment adequacy, preceding the consensus reference standard, the κ value was 0.33. Findings from the sensitivity analyses using other reference standards were consistent with the main analysis (eTable 2 in the Supplement).

**Table 1.** Characteristics of the Studied Patients (N = 302)

Characteristic	No. (%)
Age, median (IQR), y	74 (69-81)
Sex	
Female	178 (59)
Male	124 (41)
Multidose drug dispensing	33 (11)
Nursing home resident	31 (10)
No. of drugs, median (IQR)	
Regular	5 (3-7)
Regular and as needed	6 (3-9)
Top 10 drugs	
Acetaminophen	127 (42)
Omeprazole	72 (24)
Aspirin, low dose	68 (23)
Atorvastatin	68 (23)
Metoprolol	61 (20)
Cyanocobalamin	60 (20)
Furosemide	57 (19)
Simvastatin	54 (18)
Felodipine	52 (17)
Metformin	50 (17)

**Table 2.** Areas Under the ROC Curve for the 3 Sets of PIM/PPO Criteria

Criteria	Median No. of PIMs/PPOs or drugs (range)	Area under ROC curve (95% CI)	Optimal cutoff point^a^	Diagnostic measure at the optimal cutoff point					
				Sensitivity	Specificity	PPV	NPV	LR+	LR−
**PIM/PPO**									
STOPP/START									
All	1 (0-8)	0.60 (0.53-0.66)	≥2	0.37	0.79	0.46	0.72	1.74	0.80
Subset^b^	0 (0-6)	0.55 (0.48-0.62)	≥1	0.40	0.71	0.39	0.71	1.35	0.85
Swedish set	1 (0-12)	0.73 (0.67-0.80)	≥1	0.79	0.61	0.49	0.86	2.03	0.35
**PIM**									
STOPP									
All	0 (0-5)	0.56 (0.49-0.63)	≥1	0.41	0.70	0.40	0.71	1.37	0.84
Subset^b^	0 (0-5)	0.55 (0.48-0.62)	≥1	0.37	0.72	0.39	0.70	1.31	0.88
EU(7)-PIM list	1 (0-6)	0.69 (0.63-0.75)	≥1	0.79	0.54	0.45	0.84	1.71	0.40
Swedish set	0 (0-10)	0.70 (0.63-0.76)	≥1	0.68	0.66	0.49	0.81	2.02	0.48
**PPO**									
START									
All	1 (0-5)	0.57 (0.50-0.63)	≥1	0.67	0.44	0.36	0.74	1.19	0.75
Subset^b^	0 (0-4)	0.50 (0.43-0.57)	≥2	0.03	1.00	0.75	0.68	6.24	0.97
Swedish set	0 (0-3)	0.57 (0.50-0.64)	≥1	0.22	0.91	0.55	0.71	2.54	0.85
**No. of drugs^c^**									
Regular	5 (0-17)	0.69 (0.63-0.76)	≥6	0.64	0.68	0.49	0.80	1.99	0.53
Regular and as needed	6 (0-20)	0.71 (0.65-0.78)	≥7	0.71	0.67	0.51	0.83	2.14	0.43

Abbreviations: EU, European; LR−, negative likelihood ratio; LR+, positive likelihood ratio; NPV, negative predictive value; PIM, potentially inappropriate medication; PPO, potential prescribing omission; PPV, positive predictive value; ROC, receiver operating characteristic; START, Screening Tool to Alert to Right Treatment; STOPP, Screening Tool of Older Persons’ Prescriptions.

^a^ Defined as the highest sum of sensitivity and specificity.

^b^ Excluding implicit STOPP criteria: any drug prescribed without an evidence-based clinical indication and any drug prescribed beyond the recommended duration, as well as START criteria related to influenza and pneumococcal vaccinations.

^c^ Results based on the number of drugs in the medication list are presented for comparisons.

**Figure.**
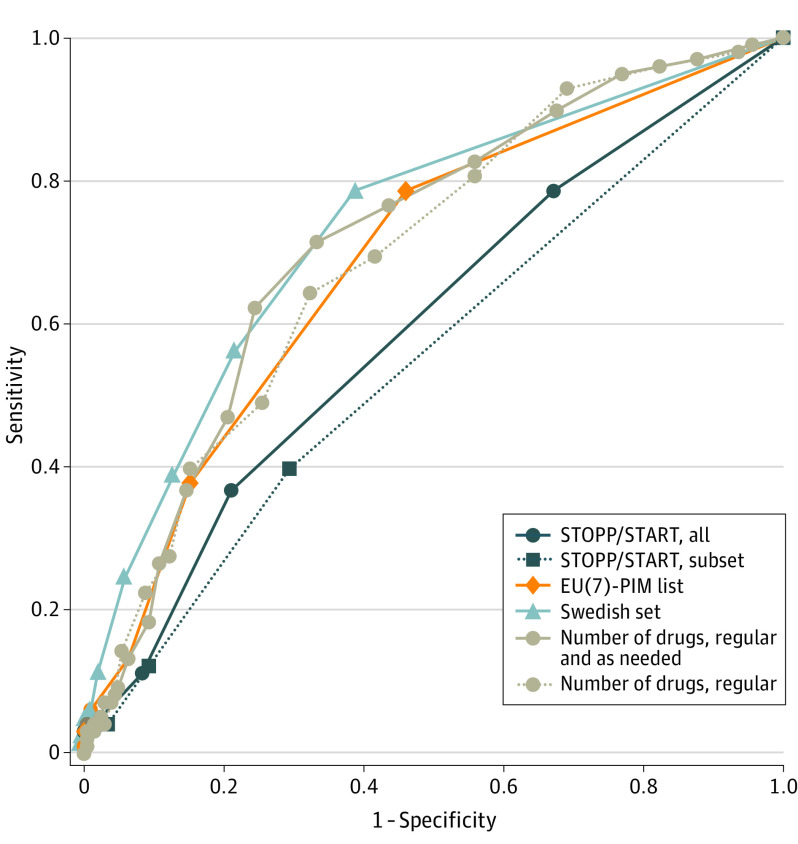
Receiver Operating Characteristic (ROC) Curves for Potentially Inappropriate Medication (PIM) and Potential Prescribing Omission (PPO) Criteria Sets to Identify Inadequate Drug Treatment PIMs/PPOs concordantly identified by 2 specialist physicians were included, with cutoff points ranging from 0 to 8 (Screening Tool of Older Persons’ Prescriptions [STOPP], Screening Tool to Alert to Right Treatment [START], all), 0 to 6 (STOPP/START, subset), 0 to 6 (European [EU][7]-PIM list), 0 to 12 (Swedish set), 0 to 20 (regular and as-needed drugs), and 0 to 17 (regular drugs). In subset analyses, implicit STOPP criteria were excluded: any drug prescribed without an evidence-based clinical indication and any drug prescribed beyond the recommended duration, as were START criteria related to use of influenza and pneumococcal vaccinations. For comparison, ROC curves based on the number of drugs in the medication list are included.

The optimal cutoff point to identify adequacy of drug treatment for concordantly identified PIMs and/or PPOs was greater than or equal to 1 for most criteria sets (Table 2). Using these cutoff points, inadequate drug treatment was correctly identified in 37% of the patients with the STOPP/START criteria, 79% with the EU(7)-PIM list, and 79% with the Swedish set. Conversely, inadequate drug treatment (1 − specificity) was incorrectly identified in 21% of the patients with the STOPP/START criteria, 46% with the EU(7)-PIM list, and 39% with the Swedish set. Using the number of drugs in the medication list to identify adequacy of drug treatment, the area under the ROC curve was 0.71 (95% CI, 0.65-0.78) (Table 2). The optimal cutoff point to discriminate between adequate and inadequate drug treatment was greater than or equal to 7, resulting in 71% of those with inadequate drug treatment being correctly identified and 33% being incorrectly identified as having inadequate drug treatment.

A total of 1140 PIMs/PPOs were identified by one assessor but not the other. The sensitivity analyses based on PIMs/PPOs identified by either of the assessors revealed areas under the ROC curve ranging from 0.71 (95% CI, 0.65-0.77; EU[7]-PIM list) to 0.77 (95% CI, 0.72-0.82; Swedish set) for the full sets (eTable 3, eFigure in the Supplement). When the cutoff points were predetermined to be greater than or equal to 1 PIM/PPO, the sensitivity varied considerably between the criteria sets and subsets; from 0.05 to 0.79 for concordantly identified PIMs/PPOs, and from 0.55 to 0.97 when all identified PIMs/PPOs were included (eTable 4 in the Supplement).

## Discussion

Evaluating the performance of established PIM/PPO criteria sets in older primary care patients, we found that the criteria have limited ability to identify inadequate drug treatment. Including PIMs/PPOs concordantly identified by 2 assessors, the full set of STOPP and START criteria displayed poor performance as a diagnostic tool, and when evaluated separately or restricted to explicit criteria not related to vaccines, the 95% CIs suggest that these criteria cannot discriminate between adequate and inadequate drug treatment. The EU(7)-PIM list and the full Swedish set, as well as the Swedish set restricted to PIMs, however, performed fairly. A simple count of the number of drugs in the medication list outperformed the STOPP/START criteria when restricted to explicit criteria not related to vaccines, the STOPP criteria, the START criteria, and the Swedish PPO criteria.

No PIM/PPO criteria set outperformed the number of drugs in the medication list as an indicator of adequacy of drug treatment. Potential explanations for this include that a substantial proportion of PIMs/PPOs are reportedly not clinically relevant for all patients.^16,17^ A recent study reported that 15% of concordantly identified PIMs/PPOs were assessed as clinically relevant, and only half of these were prioritized for medical action.^16^ This limited clinical relevance of PIMs/PPOs at the patient level is supported by the low implementation of advice provided in computerized decision support systems based on such criteria.^31^ Furthermore, another previous study reported validity problems for drug-specific indicators of prescribing quality.^32^ Given that number of drugs has been shown to be a fairly good surrogate for disease burden,^33^ it may be speculated that patients having many drugs in their medication list require more complex medical considerations, which, in turn, may be reflected in a greater number of PIMs/PPOs. This speculation is supported by the fact that the most frequent action required when treatment was considered inadequate concerned the need to set aside time to search for medical information to be able to make a decision regarding the initiation or withdrawal of a specific drug.^24^

Because our results indicate that these PIM/PPO criteria sets have limited value as indicators of adequacy of drug treatment, the suggestion to include them in core outcome sets for the evaluation of interventions for improved prescribing^7,8,9^ needs reconsideration. All examined cutoff points lacked sufficient sensitivity and/or specificity. Therefore, applying these indicator sets will unavoidably misclassify a substantial proportion of patients with inadequate as well as adequate drug treatment.

The full Swedish set showed the best balance between sensitivity and specificity. Including concordantly identified PIMs/PPOs with a cutoff point of greater than or equal to 1, 8 of 10 individuals with inadequate drug treatment would be correctly identified using this set, as would 6 of 10 with adequate drug treatment. In a previous study including drug-specific criteria from this set only, the sensitivity was lower,^32^ suggesting that extensive criteria sets are needed to achieve an acceptable sensitivity. Including concordantly identified PIMs/PPOs and using a cutoff point of greater than or equal to 1, the full STOPP/START criteria and the EU(7)-PIM list achieved similar sensitivity as the full Swedish set. The specificity for the full STOPP/START criteria, however, was low.

In the sensitivity analyses, including all PIMs/PPOs that were identified by either assessor (ie, discordant and concordant identification), the performance of the full STOPP/START criteria improved, as did the STOPP/START subset restricted to explicit criteria not related to vaccines, representing fair diagnostic tools. Using this approach, the performance of the other criteria sets moved in the same direction, but the 95% CIs overlapped. Still, no set significantly outperformed the number of drugs in the medication list as an indicator of inadequate drug treatment. The initial ambition of creating indicators of prescribing quality to assess the quality and change in quality of provided health care^3^ therefore does not seem to be fulfilled. Hence, the inclusion of PIMs/PPOs in core outcome sets for the evaluation of interventions for improved prescribing may add little value over that of simply looking at the number of concomitant medications.

Our results suggest that the EU(7)-PIM list may be more robust than the STOPP/START criteria and the Swedish set; the difference between concordantly and discordantly identified PIMs was fairly small. Because the EU(7)-PIM list includes PIMs only and does not specify the context in which a specific drug is considered potentially inappropriate, this criteria set may be less subjected to reliability issues. Comparing the effort to achieve PIM/PPO data, it must also be acknowledged that the EU(7)-PIM list is substantially less time-consuming to apply. However, recommendations evolve and some of the drugs on the EU(7)-PIM list are not generally considered inappropriate today, for instance, apixaban.

Although the present study shows that PIMs/PPOs according to these criteria sets are not valid as outcome measures, the tools reflecting experts’ opinion on drug treatment in older people may deserve further attention as pedagogic resources in medical school as well as in continuing medical education. Use for educational purposes was briefly mentioned in the recommendations from the pivotal meeting on indicators of prescribing quality in 2004.^3^ Furthermore, their value, as well as feasibility, as decision support to the prescribing physician may be worth further evaluation. The STOPP/START criteria were originally designed as an easy-to-apply and time-efficient tool for physicians to use in day-to-day practice to appraise an older patient’s prescription drugs in the context of their diagnoses, rather than as a diagnostic tool.^34^

### Strengths and Limitations

The most important strength of this study is that it provides knowledge regarding the performance of 3 established PIM/PPO criteria sets as diagnostic tools to identify inadequate drug treatment in older patients. Such evidence has hitherto been lacking in the scientific literature, although studies on concurrent validity were requested already at the initial expert meeting.^3^ Another strength of the present study is the thorough assessments of the drug treatment, with 2 specialist physicians with in-depth knowledge and experience first independently and then jointly examining cases. Nonetheless, all assessments were based on information in the medical records and important aspects, for instance, regarding patients’ preferences, may not be documented. Furthermore, it must be acknowledged that drug treatment is only one aspect of quality of care. An additional strength is that the assessments were performed in the context of another study and that the assessors did not have knowledge of the focus of the present study. This approach minimizes the risk of assessment bias. The results may also be acceptably generalizable: consecutive patients were analyzed, and the finding that most patients had PIMs and/or PPOs is consistent with recent findings in a large European study.^22^ Nevertheless, prescribing practices may differ within and between countries, and variability may have implications for the performance of the criteria sets.

This study has limitations. A substantial limitation is the lack of an established standard for characterizing the adequacy of drug treatment at the patient level. However, sensitivity analyses using other reference standards, requiring, on the one hand, the extreme that both assessors had to independently consider the drug treatment inadequate and, on the other, that merely one assessor had to make such an assessment, showed similar results as the main analysis. These analyses suggest that the results are robust. Our approach to defining the reference standard, ie, a consensus decision by 2 experienced specialist physicians preceded by independent assessments and screening using the 3 PIM/PPO sets, makes it likely that we captured medically relevant problems related to pharmacotherapy. The approach also reflects clinical work in primary care where an overall perspective is applied and medical priorities have to be made. Nevertheless, because the drug treatment was considered inadequate in cases in which a search for information was needed before decision-making, the sensitivity to identify suboptimal drug treatment may be overestimated and the specificity underestimated.

The fact that many PIMs/PPOs were discordantly identified may be regarded as a limitation. This finding is elaborated on in a separate publication.^35^ By contrast, early reliability studies of the STOPP/START criteria showed strong to almost perfect interrater identification.^36,37^ Another limitation is that the Swedish set is primarily intended for patients aged 75 years or older, whereas the STOPP/START criteria as well as the EU(7)-PIM list are intended for people 65 years or older. These considerations notwithstanding, the latter criteria sets did not outperform the Swedish set.

## Conclusions

Evaluating PIM/PPO sets as diagnostic tools to identify inadequate drug treatment, we found the performance of the full sets to range between poor and fair, comparable with a simple count of the number of drugs in the medication list. Therefore, the value of PIMs/PPOs as indicators of drug treatment quality for inclusion in core outcome sets to evaluate interventions for improved prescribing seems questionable. In future research, the value of PIM/PPO sets as pedagogic resources deserves further attention.

## Notes

Supplement. **eTable 1.** PIMs and PPOs Frequently Identified in 302 Consecutive Older Primary Care Patients **eTable 2.** Areas Under the ROC Curve According to Definition of Reference Standard **eTable 3.** Number of PIMs/PPOs per Patient, Identified by at Least 1 of 2 Specialist Physicians, and the Performance of the Three Sets of PIM/PPO Criteria in Discriminating Between Adequate and Inadequate Drug Treatment **eTable 4.** Sensitivity and Specificity, as well as Positive and Negative Predictive Values **eFigure.** ROC Curves for PIM/PPO Criteria Sets to Identify Inadequate Drug Treatment
