# Supplementary material for: Performance of 3 Sets of Criteria for Potentially Inappropriate Prescribing in Older People to Identify Inadequate Drug Treatment
Source: JAMA Netw Open. 2022 Oct 20;5(10):e2236757. doi: 10.1001/jamanetworkopen.2022.36757 (PMC9585423; doi:10.1001/jamanetworkopen.2022.36757)
Supplement: Supplement. — eTable 1. PIMs and PPOs Frequently Identified in 302 Consecutive Older Primary Care Patients eTable 2. Areas Under the ROC Curve According to Definition of Reference Standard eTable 3. Number of PIMs/PPOs per Patient, Identified by at Least 1 of 2 Specialist Physicians, and the Performance of the Three Sets of PIM/PPO Criteria in Discriminating Between Adequate and Inadequate Drug Treatment eTable 4. Sensitivity and Specificity, as well as Positive and Negative Predictive Values eFigure. ROC Curves for PIM/PPO Criteria Sets to Identify Inadequate Drug Treatment [file jamanetwopen-e2236757-s001.pdf]

Supplementary Online Content

Wallerstedt SM, Svensson SA, Lönnbro J, et al. Performance of 3 sets of criteria for potentially inappropriate prescribing in older people to identify inadequate drug treatment. *JAMA Netw Open*. 2022;5(10):e2236757. doi:10.1001/jamanetworkopen.2022.36757

eTable 1. PIMs and PPOs Frequently Identified in 302 Consecutive Older Primary Care Patients

eTable 2. Areas Under the ROC Curve According to Definition of Reference Standard

eTable 3. Number of PIMs/PPOs per Patient, Identified by at Least 1 of 2 Specialist Physicians, and the Performance of the Three Sets of PIM/PPO Criteria in Discriminating Between Adequate and Inadequate Drug Treatment

eTable 4. Sensitivity and Specificity, as well as Positive and Negative Predictive Values

eFigure. ROC Curves for PIM/PPO Criteria Sets to Identify Inadequate Drug Treatment

This supplementary material has been provided by the authors to give readers additional information about their work.

© 2022 Wallerstedt SM et al. *JAMA Network Open.*

eTable 1 PIMs and PPOs frequently identified in 302 consecutive older primary care patients (A), common medically justified actions in the 98 patients with inadequate drug treatment (B), and drugs figuring most often on these occasions (C)

A: Common PIMs/PPOs

		n (% of 302 patients)
PIMs	PPIs >8 weeks or without an evidence-based clinical indication	76 (25)
	Hypnotic Z-drugs or zopiclone >3.75 mg/d or zolpidem >5 mg/d or other drugs for insomnia including propiomazine but not benzodiazepines	58 (19)
	Presence of benzodiazepines or use of benzodiazepines >4 weeks or use of a long-acting benzodiazepine, e.g. diazepam	21 (7)
	Weak opioids, e.g. codeine or codeine >2 weeks or tramadol	17 (6)
	COX inhibitors >2 weeks or naproxen >500 mg/d or naproxen >1 week or use of diclofenac or etoricoxib	14 (5)
	Loop diuretic without a clinical indication	14 (5)
	ASA for primary prevention of cardiovascular disease	10 (3)
PPOs	Seasonal trivalent influenza vaccine annually	172 (57)
	Beta-blocker for chronic ischaemic heart disease	16 (5)
	Statin for chronic ischaemic heart disease	14 (5)
	ACE inhibitor or ARB and/or dihydropyridine calcium channel blocker and/or thiazide diuretic for hypertension	12 (4)
	Pneumococcal vaccine at least once after 65 years of age	9 (3)

B: Medically justified actions in inadequate treatment

	n (% of 182 actions)
Search for additional information in the medical records to be able to make a decision regarding the initiation or withdrawal of a specific drug	53 (29)
Withdrawing a specific drug	41 (23)
Ordering a laboratory test	25 (14)

C: Drugs figuring most often in inadequate treatment

	n (% of those treated)
PPI	18 (20)
COX inhibitors	17 (52)
Furosemide	13 (23)

ACE, angiotensin-converting enzyme; ARB, angiotensin II receptor blocker; ASA, acetylsalicylic acid; COX, cyclooxygenase; PIM, potentially inappropriate medication; PPI, proton pump inhibitor; PPO, potential prescribing omission.

Adapted from:

- *Parodi López N, Svensson SA, Wallerstedt SM. Clinical relevance of potentially inappropriate medications and potential prescribing omissions according to explicit criteria – a validation study. Eur J Clin Pharmacol 2022;78(8):1331-39*
- *Parodi López N, Svensson SA, Wallerstedt SM. Association between recorded medication reviews in primary care and adequate drug treatment management – a cross-sectional study. Scand J Prim Health Care 2021;39(4):419-428*

© 2022 Wallerstedt SM et al. *JAMA Network Open.*

eTable 2 Areas under the ROC curve according to definition of reference standard. The results were based on the number of concordantly identified PIMs/PPOs as well as the number of drugs in the medication list. Underlined figures denote that the lower 95% CI is >0.50.

			Main analysis	Sensitivity analyses	
Reference standard, inadequate drug treatment according to…			…two specialist physicians in consensus	…two specialist physicians (concordant independent assessments)	…one or two specialist physicians (concordant/ discordant independent assessments)
Patients with inadequate drug treatment, n (%)			98 (32)	53 (18)	107 (35)
PIM/PPO criteria	STOPP/START	All	0.60 (0.53–0.66)	0.57 (0.48–0.66)	0.59 (0.52–0.65)
		Subset[a]	0.55 (0.48–0.62)	0.54 (0.45–0.63)	0.56 (0.49–0.62)
	Swedish set		0.73 (0.67–0.80)	0.71 (0.64–0.79)	0.75 (0.70–0.81)
PIM criteria	STOPP	All	0.56 (0.49–0.63)	0.55 (0.46–0.64)	0.56 (0.49–0.63)
		Subset[a]	0.55 (0.48–0.62)	0.52 (0.44–0.61)	0.55 (0.48–0.61)
	EU(7)-PIM list		0.69 (0.63–0.75)	0.67 (0.59–0.74)	0.70 (0.64–0.76)
	Swedish set		0.70 (0.63–0.76)	0.67 (0.59–0.75)	0.71 (0.65–0.77)
PPO criteria	START	All	0.57 (0.50–0.63)	0.53 (0.44–0.62)	0.55 (0.48–0.62)
		Subset[a]	0.50 (0.43–0.57)	0.51 (0.43–0.60)	0.51 (0.44–0.58)
	Swedish set		0.57 (0.50–0.64)	0.58 (0.49–0.67)	0.57 (0.50–0.64)
Number of drugs	Regular		0.69 (0.63–0.76)	0.64 (0.55–0.73)	0.71 (0.65–0.77)
	Regular and as needed		0.71 (0.65–0.78)	0.68 (0.59–0.77)	0.73 (0.67–0.79)

CI, confidence interval; PIM, potentially inappropriate medication; PPO, potential prescribing omission; ROC, receiver operating characteristic; START, Screening Tool to Alert to Right Treatment; STOPP, Screening Tool of Older Persons' Prescriptions.
[a]Excluding implicit STOPP criteria: (i) any drug prescribed without an evidence-based clinical indication, and (ii) any drug prescribed beyond the recommended duration, as well as START criteria related to influenza and pneumococcal vaccinations.

© 2022 Wallerstedt SM et al. *JAMA Network Open.*

eTable 3 Number of PIMs/PPOs per patient, identified by at least one of two specialist physicians, and the performance of the three sets of PIM/PPO criteria in discriminating between adequate and inadequate drug treatment, presented as the area under the ROC curve as well as other diagnostic measures at the optimal cutpoint. Results based on the number of drugs in the medication list are presented for comparisons.

			Median number of PIMs/PPOs or drugs (range)	Area under ROC curve (95% CI)[b]	Optimal cutpoint[c]	Diagnostic measure at the optimal cutpoint					
						Sensitivity	Specificity	PPV	NPV	LR+	LR-
PIM/PPO criteria	STOPP/START	All	2 (0–15)	0.76 (0.70–0.82)	≥3	0.77	0.66	0.52	0.85	2.23	0.36
		Subset[a]	1 (0–13)	0.72 (0.67–0.78)	≥1	0.90	0.50	0.46	0.91	1.78	0.21
	Swedish set		2 (0–20)	0.77 (0.72–0.82)	≥3	0.79	0.67	0.53	0.87	2.36	0.32
PIM criteria	STOPP	All	1 (0–7)	0.73 (0.67–0.79)	≥2	0.62	0.74	0.54	0.80	2.40	0.51
		Subset[a]	1 (0–9)	0.68 (0.62–0.75)	≥1	0.73	0.59	0.46	0.82	1.80	0.45
	EU(7)-PIM list		1 (0–7)	0.71 (0.65–0.77)	≥1	0.83	0.51	0.45	0.86	1.69	0.34
	Swedish set		2 (0–17)	0.78 (0.72–0.83)	≥2	0.84	0.61	0.51	0.89	2.16	0.27
PPO criteria	START	All	1 (0–11)	0.67 (0.60–0.73)	≥2	0.58	0.70	0.48	0.78	1.95	0.60
		Subset[a]	0 (0–9)	0.63 (0.56–0.70)	≥1	0.55	0.69	0.46	0.76	1.76	0.65
	Swedish set		0 (0–7)	0.63 (0.56–0.70)	≥1	0.57	0.68	0.46	0.77	1.79	0.63
Number of drugs	Regular		5 (0–17)	0.69 (0.63–0.76)	≥6	0.64	0.68	0.49	0.80	1.99	0.53
	Regular and as needed		6 (0–20)	0.71 (0.65–0.78)	≥7	0.71	0.67	0.51	0.83	2.14	0.43

CI, confidence interval; LR-, negative likelihood ratio; LR+, positive likelihood ratio; PV, negative predictive value; PIM, potentially inappropriate medication; PPO, potential prescribing omission; PPV, positive predictive value; ROC, receiver operating characteristic; START, Screening Tool to Alert to Right Treatment; STOPP, Screening Tool of Older Persons' Prescriptions.
[a]Excluding implicit STOPP criteria: (i) any drug prescribed without an evidence-based clinical indication, and (ii) any drug prescribed beyond the recommended duration, as well as START criteria related to influenza and pneumococcal vaccinations.
[b]Underlined figures denote that the lower 95% CI is >0.50.
[c]Defined as the highest sum of sensitivity and sensitivity.

© 2022 Wallerstedt SM et al. *JAMA Network Open.*

eTable 4 Sensitivity and specificity, as well as positive and negative predictive values, of ≥1 PIM and/or PPO within each set of criteria concordantly identified by both assessors (A) or by at least one of the assessors (B), as well as ≥5 drugs in the medication list, to identify inadequate drug treatment

			Sensitivity		Specificity		PPV		NPV	
			A	B	A	B	A	B	A	B
PIM/PPO criteria	STOPP/START	All	0.79	0.97	0.33	0.22	0.36	0.37	0.76	0.94
		Subset[a]	0.40	0.90	0.71	0.50	0.39	0.46	0.71	0.91
	Swedish set		0.79	0.96	0.61	0.42	0.49	0.44	0.86	0.96
PIM criteria	STOPP	All	0.41	0.85	0.70	0.49	0.40	0.44	0.71	0.87
		Subset[a]	0.37	0.73	0.72	0.59	0.39	0.46	0.70	0.82
	EU(7)-PIM list		0.79	0.83	0.54	0.51	0.45	0.45	0.84	0.86
	Swedish set		0.68	0.93	0.66	0.49	0.49	0.46	0.81	0.93
PPO criteria	START	All	0.67	0.86	0.44	0.32	0.36	0.38	0.74	0.83
		Subset[a]	0.05	0.55	0.94	0.69	0.29	0.46	0.67	0.76
	Swedish set		0.22	0.57	0.91	0.68	0.55	0.46	0.71	0.77
≥5 drugs	Regular		0.69		0.58		0.44		0.80	
	Regular and as needed		0.83		0.44		0.42		0.84	

NPV, negative predictive value; PIM, potentially inappropriate medication; PPO, potential prescribing omission; PPV, positive predictive value; START, Screening Tool to Alert to Right Treatment; STOPP, Screening Tool of Older Persons' Prescriptions.
[a]Excluding implicit STOPP criteria: (i) any drug prescribed without an evidence-based clinical indication, and (ii) any drug prescribed beyond the recommended duration, as well as START criteria related to influenza and pneumococcal vaccinations.

© 2022 Wallerstedt SM et al. *JAMA Network Open.*

eFigure. ROC curves for PIM/PPO criteria sets to identify inadequate drug treatment

PIMs/PPOs identified by at least one of two specialist physicians were included, with cutpoints ranging from 0 to 8 (STOPP/START, all), 0 to 6 (STOPP/START, subset), 0 to 6 (EU(7)-PIM list), 0 to 12 (Swedish set), 0 to 20 (regular and as needed drugs), and 0 to 17 (regular drugs). For comparison, ROC curves based on the number of drugs in the medication list are included.

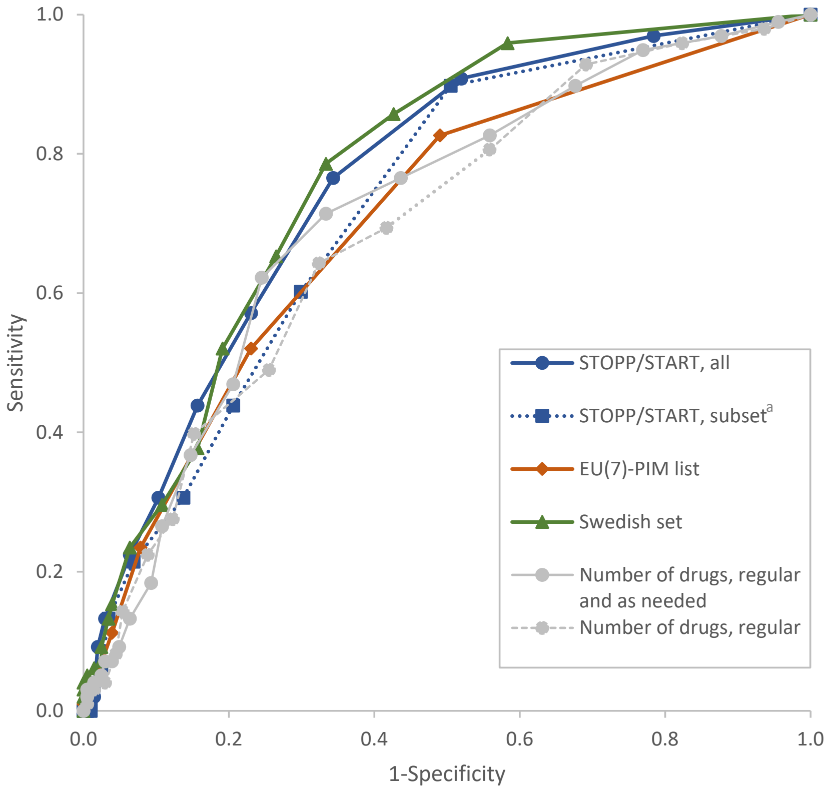

PIM, potentially inappropriate medication; PPO, potential prescribing omission; ROC, receiver operating characteristic; START, Screening Tool to Alert to Right Treatment; STOPP, Screening Tool of Older Persons' Prescriptions.

[a]Excluding implicit STOPP criteria: (i) any drug prescribed without an evidence-based clinical indication, and (ii) any drug prescribed beyond the recommended duration, as well as START criteria related to influenza and pneumococcal vaccinations.

© 2022 Wallerstedt SM et al. *JAMA Network Open.*
